# Non-contrast dual-energy CT using X-map for acute ischemic stroke: region-specific comparison with simulated 120-kVp CT and diffusion-weighted MR images

**DOI:** 10.1007/s11604-023-01490-3

**Published:** 2023-09-26

**Authors:** Yuki Shinohara, Tomomi Ohmura, Masanobu Ibaraki, Toshihide Itoh, Fumiaki Sasaki, Yuichiro Sato, Takato Inomata, Kanata Anbo, Toshibumi Kinoshita

**Affiliations:** 1 Department of Radiology and Nuclear Medicine, Research Institute for Brain and Blood Vessels-Akita, 6-10 Senshu-Kubota-Machi, Akita, 010-0874 Japan; 2 CT Research and Collaboration Department, Diagnostic Imaging Division, Siemens Healthcare K.K, Gate City Osaki West Tower 1-11-1 Osaki, Shinagawa-Ku, Tokyo, 141-8644 Japan

**Keywords:** Brain, Non-contrast CT, Dual-energy CT, Acute ischemic stroke, Diffusion-weighted MR imaging

## Abstract

**Purpose:**

X-map is a non-contrast dual-energy CT (DECT) application to identify acute ischemic stroke (AIS). Our aim was to verify region-specific characteristics of early ischemic changes (EIC) on X-map compared with simulated 120-kVp mixed-CT image and DWI.

**Methods:**

Fifty AIS patients who underwent DECT and DWI were enrolled (mean age, 76 years; 34 men, 16 women). All datasets including mixed-CT image, X-map, and DWI were transformed into a standard brain atlas with 11 × 2 ROIs based on the ASPECTS + W system. ROIs with EIC on DWI, mixed-CT image, and X-map were defined as DWI-positive, mixed-CT-positive, and X-map-positive, and those with normal finding were DWI-negative, mixed-CT-negative, and X-map-negative respectively, in visual assessment by two neuroradiologists in consensus.

**Results:**

EIC on X-maps were visually relevant to those on the other images: of 221 ROIs with mixed-CT-positive and X-map-positive, 198 (89.6%) were DWI-positive. X-map revealed moderate diagnostic accuracy for AIS compared with DWI in ROC curve analysis (AUC = 0.732). X-map identified EIC in deep white matter more sensitively than mixed-CT image: of 15 ROIs with mixed-CT-negative and X-map-positive in W segments, 14 (93.3%) were DWI-positive. X-map often showed EIC in cortical regions that were not detected on the other images: of 67 ROIs with mixed-CT-negative and X-map-positive in I and M1-M6 segments, 47 (70.1%) were DWI-negative.

**Conclusions:**

X-map is useful to detect EIC, especially in deep white matter, and may also provide additional information in acute ischemic lesions where DWI cannot be detected.

## Introduction

The results of recent randomized controlled trials in acute ischemic stroke (AIS) with large vessel occlusion (LVO) have indicated that early interventions by intravenous injection of recombinant tissue-type plasminogen activator (IV rt-PA) and endovascular thrombectomy (EVT) can be expected to improve clinical outcome [1–5]. Prior to such reperfusion therapies, it is crucial to evaluate the extent of ischemic core and LVO using CT and/or MR imaging [1–5]. Many facilities apply non-contrast CT (NCCT) to determine whether an AIS patient is indicated for IV rt-PA because of its wide availability and speed. In addition, ischemic core estimation with diffusion-weighted MR imaging (DWI) has been clinically established as one of the standard methods to determine indications of EVT [4–6].

As CT technology has developed, dual-energy CT (DECT) has become widespread in recent years. Because mass attenuation coefficients differ depending on the type of material and on photon energy, DECT using two different X-ray energies enables the material decomposition of individual voxels [7]. Furthermore, an X-ray spectrum modulation technology that employs a selective photon shield (Tin Filter technology, Siemens Healthineers, Forchheim, Germany) can improve X-ray spectral separation, enabling accurate discrimination among materials [8].

Noguchi et al. were the first to introduce the X-map technique, which uses a diagnostic task-specific three-material decomposition algorithm (3MD) to identify acute cerebral ischemic lesions on non-contrast DECT [9]. In the X-map algorithm, a virtual gray matter image is created by subtracting the lipid component mainly contained in the white matter, which is suitable for water content assessment [9–11]. Thus, the early ischemic findings of increased water content and progression of edema can be visually assessed as low attenuation on the X-map [9–11]. Since then, the X-map has improved by applying dedicated beam hardening correction and optimized parameter [11], and its ability to detect ischemic edema was supported by a recent computer simulation study [11]. Meanwhile, qualitative, quantitative, and region-specific investigation including white matter using the X-map has not been achieved in clinical cases, with reference to DWI as the gold standard method for ischemic core estimation. We hypothesized that the X-map might have additional diagnostic potential to weighted-average simulated 120-kVp mixed-CT image and DWI in the detection of early ischemic lesions. The purpose of this study was to verify the regional characteristics of early ischemic changes (EIC) on X-map compared with weighted-average simulated 120-kVp mixed-CT image and DWI.

## Materials and methods

### Patients

**Fig. 1:**
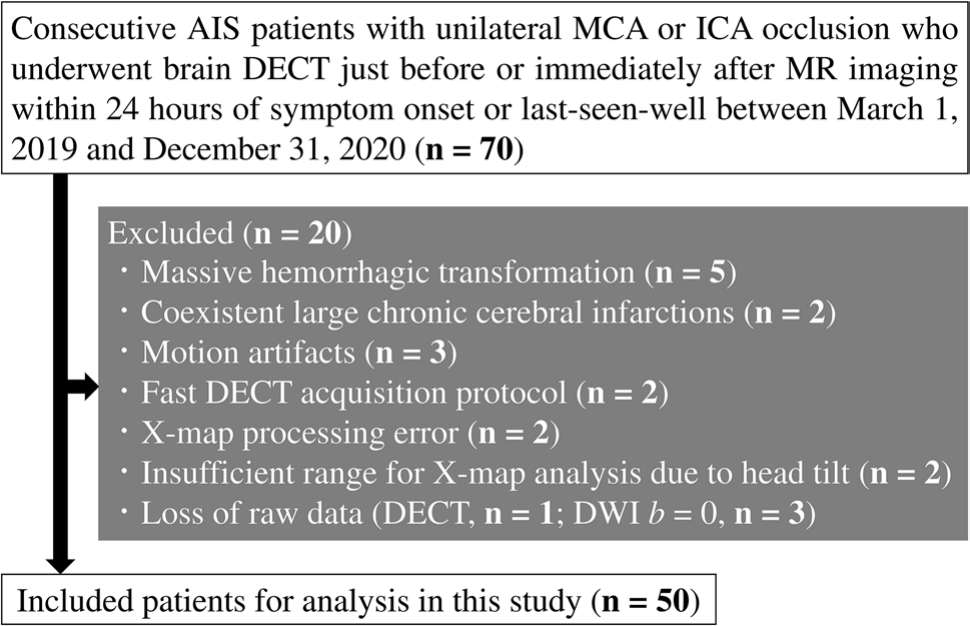
Flowchart for patient selection

The study protocol was approved by the ethics committee in our institution, and informed consent was waived because of its retrospective design. This study enrolled all AIS patients with unilateral middle cerebral artery (MCA) or internal carotid artery (ICA) occlusions who had undergone brain DECT just before or immediately after MR imaging within 24 h of symptom onset or last-known-well at our institution between March 1, 2019, and December 31, 2020. Seventy patients were enrolled, of whom 20 were excluded for the following reasons: massive hemorrhagic transformation (n = 5) and coexistent large chronic cerebral infarctions (n = 2), considering the possibility of misalignment with the standard brain in anatomical standardization; motion artifacts, n = 3; faster DECT acquisition protocol for stroke (gantry rotation, 0.5 s) than the routine one, n = 2; X-map processing error, n = 2; insufficient range for analysis of X-map due to head tilt, n = 2; loss of DWI b = 0 raw data, n = 3; and loss of DECT raw data, n = 1. A final total of 50 patients (34 males and 16 females; mean age ± standard deviation [SD], 76 ± 12 years) were included in the study (Fig. 1 and Table 1). The median time intervals between symptom onset or last-know-well and brain DECT was 137 min.

**Table 1:** Patients’ demographics and clinical characteristics

Characteristic	Baseline characteristic (n = 50)
Age (mean ± SD)	76 ± 12
Gender	
Male	34 (68)
Female	16 (32)
Laterality of large vessel occlusion	
Right	26 (52)
Left	24 (48)
Number of patients who underwent IV rt-PA only	7^a^ (14)
Number of patients who underwent EVT only	8 (16)
Number of patients who underwent both IV rt-PA and EVT	19^a^ (38)

Percentages are given in parentheses. ^a^IV rt-PA was suspended in three patients because of a large ischemic core on simultaneous MRI. Another three patients started receiving IV rt-PA in other hospitals and they were transferred to our institution by the drip-and-ship method. Then, non-contrast DECT and MR imaging of the brain were performed to determine therapeutic indication of EVT in our institution

### CT systems and protocols

Brain NCCT was performed using a dual-source CT system (SOMATOM Drive; Siemens Healthineers). The two X-ray tubes were operated at kilovoltage settings of 80 and 140 kVp using a dedicated tin filter (Sn140 kVp), and the following DECT parameters: collimation width, 40 × 0.6 mm; gantry rotation, 1.0 s; pitch, 0.7; automatic attenuation-based tube current modulation (CARE Dose 4D; Siemens Healthineers); slice thickness for reconstruction, 1.0 mm. The average volume CT dose index was 75.2 ± 5.2 mGy (range, 62.7–86.1 mGy), and the mean dose–length product was 1304.6 ± 180.4 mGy⋅cm (range, 951.7–1715.8 mGy⋅cm).

The 80 kVp and Sn140 kVp mixed-CT image was obtained with a weighting factor of 0.4 to simulate an equivalent polychromatic 120-kVp image, using a dedicated head kernel (Hf39) and an advanced modeled iterative reconstruction (ADMIRE, strength 4; Siemens Healthineers). The DE dataset was transferred to a *syngo*.via workstation (Siemens Healthineers) and stored in an image folder for the X-map processing. The stored dataset was loaded offline into a X-map package in MATLAB software (MathWorks Inc., Natick, MA, USA) to create the image. The current version of X-map applied a dedicated beam hardening correction inside the algorithm for SOMATOM Drive (Siemens Healthineers), and optimized the attenuation values of gray and white matters and the slope of their connecting line to a pair of 80 kVp/Sn140 kVp tube voltages using a dedicated parameter.

### MR imaging systems and protocols

All enrolled patients underwent brain MR imaging using a MAGNETOM Skyra 3 T (47 patients), Verio 3 T (one patient), or Aera 1.5 T MR system (two patients) (Siemens Healthineers). In our institution, DWI is used as the gold standard for determining indications of EVT in patients with AIS. The DWI scan protocols were as follows: Skyra: TR/TE, 5000/57 ms; field-of-view, 230 mm; bandwidth, 1628 Hz/pixel; slice thickness, 5 mm; acquisition time, 48 s; Verio: TR/TE, 6000/57 ms; field-of-view, 230 mm; bandwidth, 1628 Hz/pixel; slice thickness, 5 mm; acquisition time, 1 min 12 s; and Aera: TR/TE, 5500/67 ms; field-of-view, 230 mm; bandwidth, 1502 Hz/pixel; slice thickness, 5 mm; acquisition time, 57 s.

### Anatomical standardization and regions of interest analysis

**Fig. 2:**
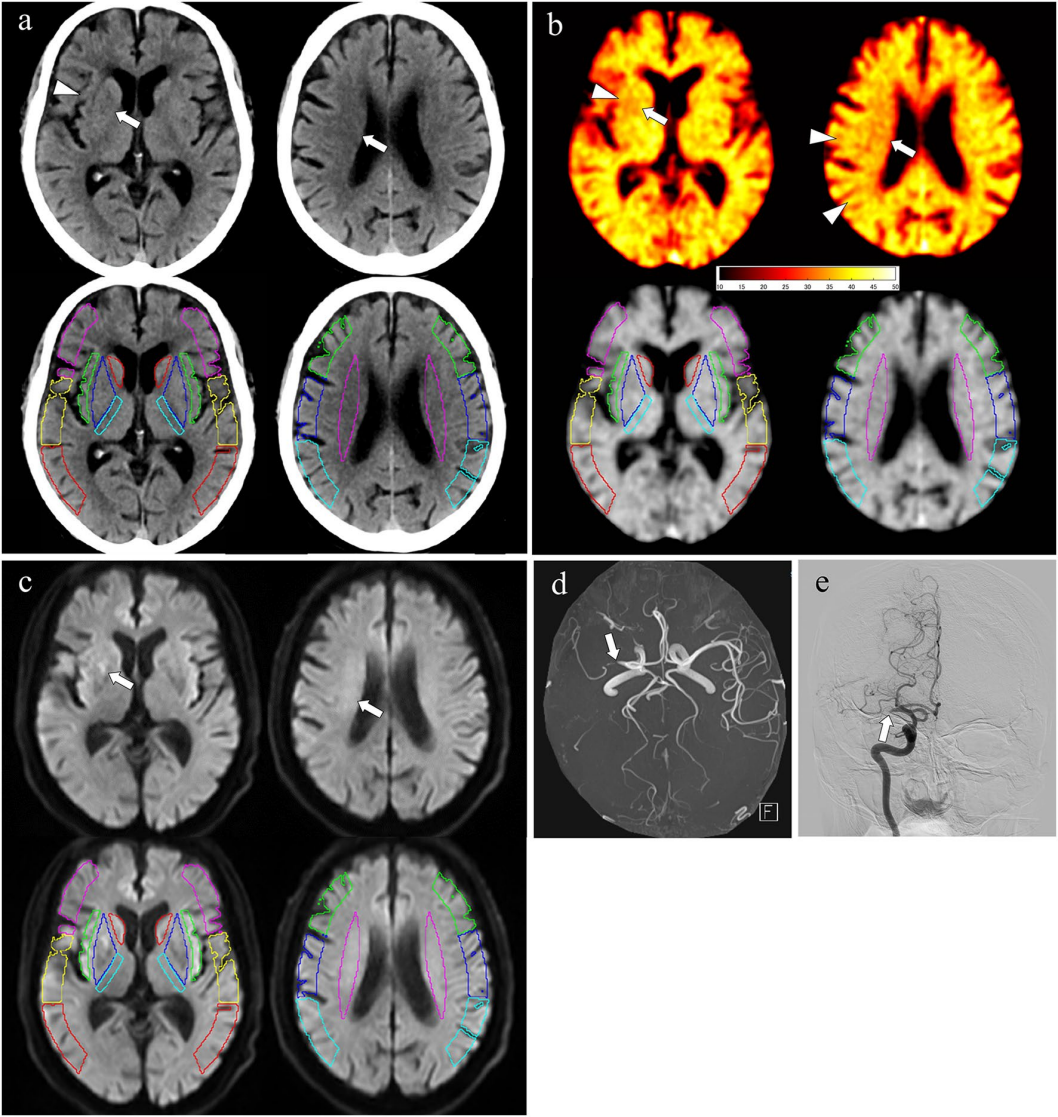
An 83-year-old male with acute right MCA occlusion. Simulated 120-kVp mixed-CT image (**a**), X-map (**b**), DWI (**c**), images with ROIs based on the ASPECTS + W system (**a–c**), MRA (**d**), and DSA (**e**, antero-posterior view) of right internal carotid artery before endovascular thrombectomy are shown. The mixed-CT image shows early ischemic changes (EIC) in the right insular cortex (I) (**a**, arrowhead), lentiform nucleus (L) (a, arrow), and deep white matter (W) (**a**, arrow). The X-map demonstrates hypoattenuation in the right cortical MCA regions of M5 and M6 (**b**, arrowheads) as well as I (**b**, arrowhead), L (**b**, arrow), and W segments (**b**, arrow). The DWI reveals high intensity in the right L and W segments (**c**, arrows). Both MRA and DSA show the right proximal MCA occlusion (**d** and **e**, arrows) with leptomeningeal collaterals, which indicates hypoperfusion in the right MCA territory. In this patient, the M5 and M6 segments are the ischemic area where only X-map shows hypoattenuation

All datasets including mixed-CT image, X-map, and DWI b = 1000 were transformed into a standard brain atlas using SPM12 (Wellcome Department of Cognitive Neurology, London, UK) [12]. Regions of interest (ROIs) were placed at the level of the basal ganglia (1 mm thickness × 7 slices) and corona radiata (1 mm thickness × 7 slices) on the standardized mixed-CT images according to the Alberta Stroke Program Early Computed Tomography Score including deep white matter (ASPECTS + W) system [13] (Fig. 2). To exclude pixels containing bone and cerebrospinal fluid from each ROI in the mixed-CT images, pixels with CT attenuation < 20 or > 80 HU were not included. The same ROI templates set on the mixed-CT images were used on the X-maps and DWIs.

### Qualitative evaluation

In DWI based on the ASPECTS + W system, each ROI was classified as either DWI-positive or DWI-negative by the consensus of two experienced neuroradiologists (YS and TK, with 18 and 30 years of experience, respectively). DWI-positive was defined as high intensity indicating an area of acute infarction, and DWI-negative was defined as isointensity indicating a non-infarcted area.

In the mixed-CT images, each ROI was classified as either mixed-CT-positive or mixed-CT-negative. Mixed-CT-positive was defined as showing EIC including blurring of gray–white matter discrimination, loss of the insular ribbon, and obscuration of the lentiform nucleus [14, 15]; mixed-CT-negative was defined as showing no evidence of EIC. The hot iron color scale was used for the visual assessment of the X-map (Fig. 2b, upper row). Since the contour of each ROI was difficult to identify in the hot iron color, the X-map using gray scale with ROIs were also prepared (Fig. 2b, lower row). Thus, the ischemic lesions on the X-map were evaluated by using hot iron color map, and those locations were referred to the ROIs on the gray scale map. The X-map findings corresponding to each ROI were classified into two groups: X-map-positive, showing low-attenuation in the X-map using hot iron color scale; and X-map-negative, showing iso-attenuation in the X-map using hot iron color scale, relative to the contralateral side and area surrounding the ROI. All interpretations of the mixed-CT images and X-maps were performed by two experienced neuroradiologists (YS and TK, as above) independently at first, followed by the consensus approach. The readers were told the affected side but were blinded to the severity of stroke.

### Quantitative evaluation

The difference in mean X-map values (HU) between affected and unaffected hemisphere was termed delta X-map values (ΔX-map). Mann–Whitney *U* test was performed to evaluate differences in ΔX-map between DWI-positive and DWI-negative. Area under the receiver-operating characteristic (ROC) curve (AUC) was used to evaluate the diagnostic accuracy of X-map for AIS based on the DWI findings. The AUC values were considered as follows: < 0.70, low diagnostic accuracy; 0.70–0.90, moderate diagnostic accuracy; and ≥ 0.90, high diagnostic accuracy. All statistical analyses were performed using SPSS version 28.0 (SPSS Inc., Chicago, IL, USA). Statistical significance was established at *P* < 0.05.

## Results

Among the 550 ROIs in the affected sides, 290 were DWI-positive and 260 were DWI-negative; in addition, 229 and 321 were mixed-CT-positive and mixed-CT-negative, respectively; and 312 and 238 were X-map-positive and X-map-negative, respectively (Table 2).

**Table 2:** Qualitative analysis among mixed-CT images, X-maps, and DWIs

	Mixed-CT/X-map match		Mixed-CT/X-map mismatch	
	mixed-CT-positive and X-map-positive	mixed-CT-negative and X-map-negative	mixed-CT-positive and X-map-negative	mixed-CT-negative and X-map-positive
DWI-positive	198 (89.6)	50 (21.7)	2 (25.0)	40 (44.0)
DWI-negative	23 (10.4)	180 (78.3)	6 (75.0)	51 (56.0)
Total	221	230	8	91

Percentages are given in parentheses

### Qualitative evaluation

The EIC on X-map were consistent with those of mixed-CT images and DWIs in many cases: of 221 ROIs that were X-map-positive and mixed-CT-positive, 198 (89.6%) were also DWI-positive; and of 230 ROIs that were X-map-negative and mixed-CT-negative, 180 (78.3%) were also DWI-negative (Table 2).

**Fig. 3:**
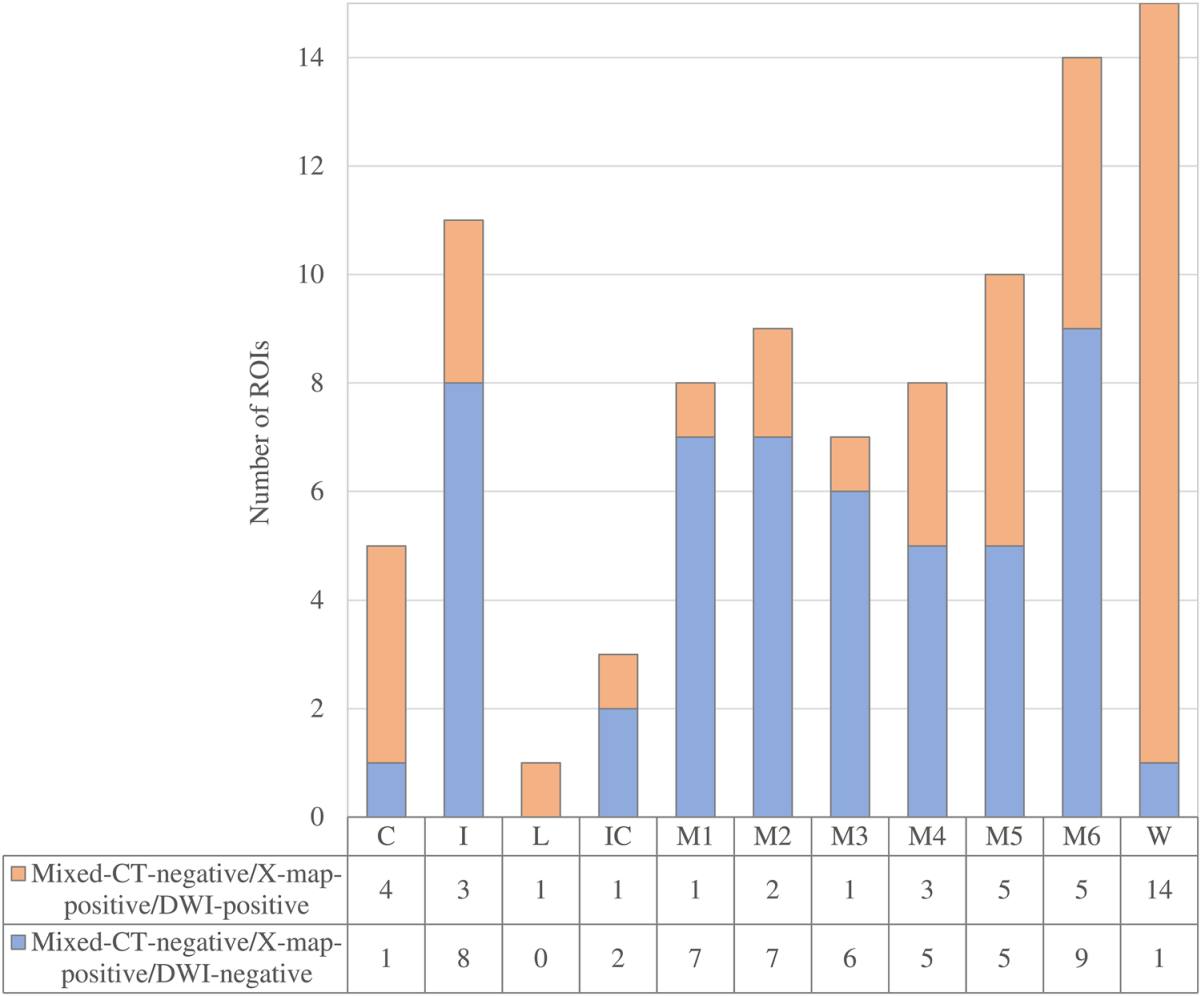
Stacked bar chart of the combination of mixed-CT-negative and X-map-positive findings in each ASPECTS + W segment. Among the mixed-CT/X-map mismatched ROIs, mixed-CT-negative, X-map-positive, and DWI-positive findings were common combination in W segment (14/40 ROIs, 35.0%), whereas mixed-CT-negative, X-map-positive, and DWI-negative findings were common combination in the insular cortex (I, 8/51 ROIs; 15.7%) and in all cortical MCA regions (M1–6, 39/51 ROIs; 76.5%)

The discrepancy between X-map and mixed-CT image, especially the combination of X-map-positive and mixed-CT-negative findings, were often seen in deep white matter (W segment) and cortical branch regions (I and M1-M6 segments) (Fig. 3).

**Fig. 4:**
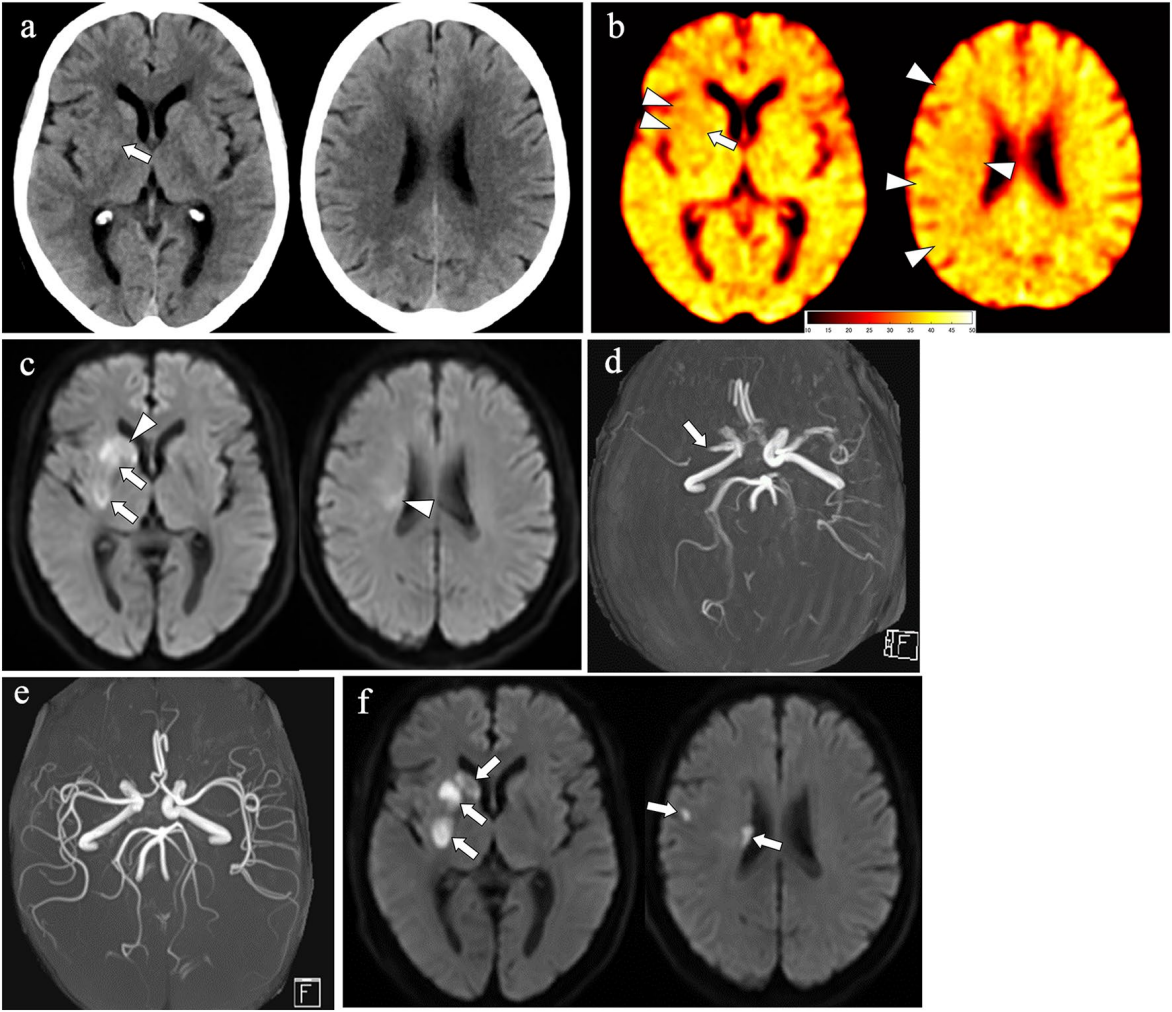
A 77-year-old female with acute right MCA occlusion. Standardized mixed-CT image, X-map, and DWI respectively reveal EIC (**a**, arrow), hypoattenuation (**b**, arrow), and hyperintensity (**c**, arrows) in the right lentiform nucleus (L) indicating acute ischemic infarction, and DWI also reveals hyperintensity in the right caudate nucleus (C) and W segments (**c**, arrowheads). On the other hand, X-map shows hypoattenuation in the right W segment (**b**, arrowhead) that is not detected on the mixed-CT image (**a**), and X-map reveals hypoattenuation in the right I, M4, M5, and M6 segments (**b**, arrowheads) that is not apparent on the mixed-CT image (**a**) and the DWI (**c**). Considering the initial MRA finding of right proximal MCA occlusion (**d**, arrow), these mismatched lesions on the X-map (**b**, arrowheads) are included in the right occluded MCA territory without restricted diffusion, i.e., MRA-DWI mismatch area. MRA after reperfusion therapy on day 1 reveals complete recanalization of the right MCA (**e**). Standardized follow-up DWI (**f**) on day 1 shows hyperintensity in the right L, C, W, and a small part of M5 segments (arrows), which indicates irreversible acute infarction. The right I, M4, M5, and M6 segments where only the X-map revealed hypoattenuation (**b**, arrowheads) are almost intact on the follow-up images (**f**). Thus, the X-map hypoattenuation in the MRA-DWI mismatch area may reflect early minimal change in water content caused by the decreased perfusion pressure, where successful reperfusion can rescue

The early ischemic findings in deep white matter can be evaluated appropriately on the X-map in comparison with the mixed-CT image: of 15 ROIs with X-map-positive and mixed-CT-negative in W segments, 14 (93.3%) showed DWI-positive (Figs. 3 and 4); and there was no ROI that was mixed-CT-positive and X-map-negative in W segments.

The combination of X-map-positive, mixed-CT-negative, and DWI-negative findings was frequently seen in the cortical branch regions: of 11 ROIs that were X-map-positive and mixed-CT-negative in I segments, 8 (72.7%) showed DWI-negative; and of 56 ROIs with X-map-positive and mixed-CT-negative in M1-M6 segments, 39 (69.6%) showed DWI-negative (Figs. 2, 3 and 4).

### Quantitative evaluation

The median ΔX-map for DWI-positive was significantly lower than that for DWI-negative in the area that combined all ASPECTS + W segments (P < 0.05) (Table 3). The median ΔX-maps for DWI-positive showed significant lower values than those for DWI-negative in C, I, L, M1, M2, M4, and M5 segments (P < 0.05 for each), whereas median ΔX-maps for DWI-positive were not statistically different from those for DWI-negative in IC (P = 0.143), M3 (P = 0.609), M6 (P = 0.065), and W segments (P = 0.326).

**Table 3:** Result of quantitative analysis for X-map

	Median ΔX-map (quartiles)			AUC (95% confidence interval)
	DWI-positive	DWI-negative	*P*-value	
All	−1.40 (−2.72, −0.29)	−0.16 (−1.05, 1.01)	< 0.05	0.732 (0.691, 0.773)
C	−1.09 (−1.77, −0.23)	0.530 (0, 2.12)	< 0.05	0.817 (0.700, 0.935)
I	−1.40 (−3.00, −0.05)	0.250 (−1.94, 1.46)	< 0.05	0.699 (0.528, 0.870)
L	−1.82 (−2.68, −1.15)	0.21 (−0.34, 0.95)	< 0.05	0.925 (0.853, 0.997)
IC	−0.985 (−1.71, −0.335)	−0.290 (−0.920, 0.353)	0.143	0.723 (0.464, 0.982)
M1	−2.275 (−3.92, −0.988)	−0.200 (−1.66, 0.873)	< 0.05	0.799 (0.677, 0.920)
M2	−1.67 (−2.89, −0.52)	0.01 (−0.80, 1.08)	< 0.05	0.736 (0.593, 0.878)
M3	−0.55 (−2.44, 0.93)	−0.38 (−1.17, 0.59)	0.609	0.543 (0.365, 0.720)
M4	−1.8 (−3.42, −0.52)	−0.3 (−1.22, 0.94)	< 0.05	0.738 (0.596, 0.880)
M5	−1.41 (−2.40, −0.833)	−0.445 (−1.01, 0.918)	< 0.05	0.763 (0.631, 0.895)
M6	−1.05 (−2.56, −0.143)	−0.405 (−1.29, 1.18)	0.065	0.653 (0.501, 0.806)
W	−0.940 (−1.75, −0.180)	−0.155 (−1.73, 1.49)	0.326	0.627 (0.347, 0.907)

In ROC curve analysis, X-map in the combined all ASPECTS + W segments showed moderate diagnostic accuracy for AIS compared with DWI (AUC = 0.732) (Table 3). The X-map revealed high diagnostic accuracy for AIS in L (AUC = 0.925) and moderate in C (AUC = 0.817), IC (AUC = 0.723), M1 (AUC = 0.799), M2 (AUC = 0.736), M4 (AUC = 0.738), and M5 segments (AUC = 0.763), whereas the X-map demonstrated low diagnostic accuracy in I (AUC = 0.699), M3 (AUC = 0.543), M6 (AUC = 0.653), and W segments (AUC = 0.627) in comparison with DWI.

## Discussion

We validated the region-specific characteristics of EIC on X-map by qualitative and quantitative evaluation using ASPECTS + W system. Results showed a high relevance of early ischemic findings on X-map to the DWI findings, especially in deep gray matter such as caudate and lentiform nuclei. The X-map also demonstrated an additive diagnostic value to mixed-CT image for the detectability of acute ischemic lesions in deep white matter. Moreover, the X-map could reveal EIC in cortical segments such as insular and cerebral cortices, where both mixed-CT image and DWI showed normal findings.

NCCT plays a key role in the accurate and rapid diagnosis of acute infarction as well as intracranial hemorrhage in stroke patients. In AIS, NCCT hypoattenuation represents physiological water uptake in irreversibly damaged brain tissue [16–18]. However, EIC on NCCT are likely to be so subtle that interpretation can vary among readers. Several methods using non-contrast DECT have recently been reported to improve the visualization of acute ischemic lesions [9, 10, 19–22]; among these, X-map is an advanced DECT application that enables identification and quantification of acute cerebral infarction using 3MD [9]. CT attenuation values differ between gray and white matter because the gray matter has relatively high water and lower lipid contents, and thus greater photoelectric absorption [23]. The lipid component mainly in the white matter is subtracted using the X-map application, resulting in a suitable image for assessing water content [9, 10]. That is, the early ischemic findings of increased water content and progression of edema can be visualized as hypoattenuation [9, 10]. According to the latest virtual phantom simulation [11], the X-map showed higher detectability of low-contrast object mimicking ischemic edema than the mixed-CT image. Since there has been no validation study using the current version of X-map in patients with AIS so far, this is the first report to verify qualitative and quantitative evaluation based on region-specific assessments using an ASPECTS + W classification using the X-map.

The present results revealed many cases of matched X-map and mixed-CT image findings corresponding with those of DWIs, which supports the reliability of X-map for identifying AIS. However, ROIs with mismatched findings among them existed to some extent. For example, with the ASPECTS + W system, 14/40 (35%) ROIs that were mixed-CT-negative, X-map-positive, and DWI-positive were found in the deep white matter. The ASPECTS + W system is based on an 11-point method, including deep white matter, to evaluate the extent of acute ischemic lesions on DWI [13]. As white matter has a lower ischemic threshold than cortex, white matter lesions on DWI are considered an imaging biomarker for resistance to thrombolysis [24]. Additionally, when comparing the predictability of intracranial hemorrhagic complications among CT-ASPECTS [25], DWI-ASPECTS [26], and ASPECTS + W [13], ASPECTS + W was the only system that could significantly predict hemorrhagic transformation [13, 27]. In AIS patients, it is therefore clinically important to perform radiological assessment of the deep white matter as well as the cortex. However, it is difficult to detect EIC in the cerebral white matter on conventional 120-kVp NCCT [25]. The present results indicate the superior accuracy of X-map compared with mixed-CT image for evaluating acute ischemic lesions in white matter on NCCT, which is the first major finding of the study. Of note, a recent computer simulation research also indicated that X-map was superior to mixed-CT image regarding the detectability of EIC in white matter [11], which can be supported by our results using clinical cases. Meanwhile, although a median ΔX-map in the deep white matter for DWI-positive was lower than that for DWI-negative in W segment, there was no statistically significant difference between them (Table 3). Since the hypoattenuation due to leukoaraiosis might have influenced the results, it is important to identify differences between EIC and leukoaraiosis by focusing on its distribution and attenuation when performing visual assessment in deep white matter on the X-map. Considering clinical use, a side-by-side comparison of mixed-CT image, which can be obtained at the same time as the X-map, may be helpful to identify EIC in white matter on the X-map.

Although the overall diagnostic accuracy for AIS on X-map was favorable in the quantitative analysis, the X-map did not necessary show high diagnostic accuracy in some ASPECTS + W segments compared with DWI (Table 3). Indeed, there were several mismatched findings in X-map versus DWI in the qualitative analysis (Table 2). A possible reason for these mismatches might be the intrinsic difference in imaging characteristics between X-map and DWI. Especially, the image noise on DE material decomposition, including X-map, is increased compared to that on single-energy conventional CT, because the DE material decomposition assumes that each voxel comprises a linear combination of target materials and its overall dose is split by each material image, resulting in increased image noise [28]. In a clinical setting, although it may be difficult to fully replace conventional NCCT to X-map as a diagnostic tool for detecting EIC, the X-map can be a diagnostic support tool of mixed-CT image and play a complementary role to mixed-CT image and/or DWI.

This study has multiple limitations. First, this is a retrospective study with relatively small sample size. Many potential subjects could not be enrolled because of the exclusion criteria. Second, no patient underwent perfusion CT or MR imaging, and not all the patients received follow-up MR imaging. One of our results showed the finding of mixed-CT-negative, X-map-positive, and DWI-negative in several ASPECTS + W segments, particularly in cortical regions. This finding was identified in vascular territories corresponding to the occluded vessels on MRA other than the relatively small hyperintensity on DWI, so-called MRA-DWI mismatch [29]. In some patients who received follow-up MR imaging, the isolated X-map hypoattenuation area appeared to be salvageable after successful reperfusion (Fig. 4). As one possible reason, the isolated X-map-positive finding may reflect early minimal change in water content due to reduced perfusion pressure. However, since it is difficult to interpret precise pathophysiology of this finding without perfusion CT or MR imaging and follow-up MR imaging, we cannot rule out the possibility that it is just a false-positive finding due to some artifact. To elucidate the pathophysiology of the X-map hypoattenuation without EIC on mixed-CT image and DWI, large-scale prospective investigations using perfusion CT or MR imaging and follow-up MR imaging are necessary to validate our results. Third, DWI was acquired using two 3 T and one 1.5 T MR scanners. Although the difference of magnetic field strengths might have affected the EIC detectability on DWI between 3 T and 1.5 T scanners [30], it would have little influence on our results because the 1.5 T scanner was used in only two cases whereas the 3 T scanners were used in most cases (48 out of 50 patients). Finally, we did not evaluate EIC on X-map outside the region of the ASPECTS + W system. X-map assessment of EIC in the whole cerebrum is required in further studies.

## Conclusions

In a region-specific assessment using the ASPECTS + W system, X-map has an additive diagnostic value to detect EIC. Especially, the X-map can demonstrate favorable detectability of acute ischemic lesions in deep white matter as well as deep gray matter. Meanwhile, isolated hypoperfusion on the X-map without EIC on mixed-CT image and DWI can be seen frequently in cortical branch region, which may indicate salvageable ischemic lesion. Further prospective investigations including perfusion CT or MR imaging and follow-up imaging and direct comparison with continuous slices are warranted to clarify its pathophysiology.
